# Synergistic Toxicity of Fine Particulate Matter and Ozone and Their Underlying Mechanisms

**DOI:** 10.3390/toxics13040236

**Published:** 2025-03-24

**Authors:** Jing He, Tong Wang, Han Li, Yemian Zhou, Yun Liu, An Xu

**Affiliations:** 1 Institutes of Physical Science and Information Technology, Anhui University, Hefei 230601, China; hj032100@163.com; 2 High Magnetic Field Laboratory, Hefei Institutes of Physical Science, Chinese Academy of Sciences, Hefei 230031, China; lhana@mail.ustc.edu.cn (H.L.); 18756020733@163.com (Y.Z.); 3 School of Public Health, Anhui University of Science and Technology, Hefei 231131, China; twang1994@aust.edu.cn; 4 Science Island Branch, Graduate School of USTC, University of Science and Technology of China, Hefei 230026, China

**Keywords:** PM_2.5_ and ozone, synergistic toxicity, mechanism

## Abstract

The co-occurrence of fine particulate matter (PM_2.5_) and ozone has emerged as a critical environmental challenge in recent years. The individual harmful impacts of PM_2.5_ and ozone exposure have been well studied; however, their combined toxicity under co-exposure conditions remains mechanistically undefined. This paper provides an extensive evaluation of the current pollution levels, epidemiological investigation, and new findings on the toxicological mechanisms of combined exposure to PM_2.5_ and ozone. The synergistic toxicity of PM_2.5_ and ozone depends on different factors, including the physicochemical properties of PM_2.5_, the dose and duration of exposure, and the specific target organs. Through extensive research, we identified the main targets of toxic responses to PM_2.5_ and ozone exposure and summarized their synergistic toxic mechanisms. Given the current research priorities, there is an urgent need to improve scientific research regarding PM_2.5_ and ozone co-exposure with priority given to characterizing their properties and toxicological responses while updating relevant guidelines and standards.

## 1. Introduction

Air pollution has become an important issue in global development and likely the most serious environmental challenge for governments and international organizations. Various kinds of air pollutants, including ozone (O_3_), carbon monoxide, nitrogen oxides, and particulate matter (PM) of different sizes, pose a serious threat to human health. Among these pollutants, fine particulate matter (PM_2.5_, i.e., particles less than or equal to 2.5 µm in diameter) and ozone are two of the most harmful air pollutants, leading to serious adverse effects on the respiratory, cardiovascular, and nervous systems. These two pollutants are regarded as major threats to public well-being and economic development [1]. In recent years, the implementation of China’s ‘Ten Rules for the Atmosphere’ policy and the ‘Three-Year Action Plan’ has significantly improved air quality [2,3]. However, the problem of secondary pollutants, which are transformed from primary pollutants (directly discharged into the environment) through chemical or photochemical reactions, has become increasingly prominent [4]. PM_2.5_ has characteristics of both primary and secondary pollutants, whereas surface ozone is a classic secondary pollutant [5,6,7,8]. Surface ozone and PM_2.5_ are two major pollutants in China [9,10,11]. PM_2.5_ and ozone have different levels of spatial association across different zones, such as urban and suburban ones. It was found that nitrogen dioxide concentrations were significantly higher in urban areas than in suburban areas, and direct emissions of PM_2.5_ co-existed with ozone precursors (NO_x_). Elevated levels of NO_x_ can rapidly deplete ozone through the ozone titration effect (O_3_ + NO → NO_2_ + O_2_), causing a negative correlation between PM_2.5_ and ozone levels. In contrast, in suburban areas where NO_x_ concentrations are lower due to the distance from direct PM_2.5_ emission sources, volatile organic compounds (VOCs) play a dominant role in the photochemical reactions and promote ozone formation whereas PM_2.5_ is primarily from secondary aerosols [12]. The combined toxicity of PM_2.5_ and ozone may be different from the toxicity of a single pollutant, and there may be synergies between them [13]. It is concerning that background ozone levels in certain regions of China are much higher than those found in some cities of developed countries [14,15]. During the ‘14th Five-Year Plan’ period, the integrated management of PM_2.5_ and ozone will become an important component of air pollution prevention and control efforts [16,17]. There is an urgent need to break away from the traditional research paradigm focusing on individual pollutants to establish a comprehensive climate-health joint warning system and it is crucial to develop precise intervention strategies aimed at pollutant metabolism and detoxification in the field of environmental medicine. The assessment necessitates a comprehensive epidemiological evaluation of the health effects of PM_2.5_ and ozone on individuals across different ages, genders, and pre-existing health conditions. Researchers need to conduct a comprehensive fundamental investigation to elucidate the synergistic toxic effects of PM_2.5_ and ozone under different conditions. It is essential to identify specific signaling pathways or biomarkers, reveal toxicity mechanisms, and provide a scientific basis for developing new intervention strategies.

This review systematically integrates the latest achievements in the field of environmental health science and reconstructs a comprehensive exposure–health effect map for PM_2.5_ and ozone through multidimensional evidence chains, such as global multicenter cohort studies, toxicology dynamic model studies, and toxicology mechanism explorations. It outlines the global distribution characteristics and the changing trends of pollutants worldwide while underscoring their significant threats to human health. By examining the potential molecular mechanisms, the review highlights the adverse health impacts caused by combined exposure to PM_2.5_ and ozone on various human systems, including the respiratory, cardiovascular, and nervous systems. This paper aims to provide solid evidence on preventing and controlling diseases caused by air pollutants and the clinical treatment of those conditions.

## 2. Current Status of PM_2.5_ and Ozone Pollution

PM_2.5_ and near-surface ozone are significant contributors to climate change and pose serious threats to human health [18]. Through the collaborative efforts of the World Health Organization (WHO) and governments around the globe, there has been a notable improvement in PM_2.5_ control measures, leading to a significant reduction in PM_2.5_ concentrations globally. The U.S. Federal Environmental Protection Agency (EPA) has updated the ‘Class I’ standard for annual average PM_2.5_ concentrations from 12 µg/m^3^ to 9 µg/m^3^, indicating higher air quality standards [19]. ‘China’s Ecological and Environmental Status Bulletin’ shows that between 2016 and 2023, the average concentration of PM_2.5_ in urban ambient air decreased from 42 µg/m^3^ to 30 µg/m^3^, achieving a significant reduction of 28.6 percent [20]. These data indicate that China has made substantial progress in the management of PM_2.5_ levels. However, the current global concentration level of PM_2.5_ still has a considerable distance to cover before reaching the WHO-recommended guideline of 5 µg/m^3^ as outlined in the Air Quality Guidelines (AQG) [21].

At the same time, the world is facing a pervasive issue of global surface ozone pollution, whose impacts are continuously expanding over a substantially long pollution season. In 2019 alone, ozone exposure caused 365,000 premature deaths worldwide [22]. In the same year, short-term exposure to PM_2.5_ and ozone caused approximately 713.5 thousand (95% Confidence Interval: 598.8–843.3) and 496.3 (371.3–646.1) thousand mortalities globally, respectively. The peak concentrations recorded were 631.2 µg/m^3^ for PM_2.5_ and 357.3 µg/m^3^ for ozone, presenting a substantial health risk challenge in South and East Asia [18]. On a global scale, an alarming trend is emerging: up to 47.5% of the cities are experiencing PM_2.5_ and ozone pollution, which is a signal of environmental degradation and a severe threat to human health [23]. According to the American Lung Association’s ‘State of the Air 2024’ report, 39% of Americans continue to live in areas affected by ozone or particulate matter pollution [24]. In China, the concurrent pollution of PM_2.5_ and ozone is becoming increasingly severe. Lyu et al. [25] conducted an extensive study in southern China, especially the Pearl River Delta (PRD) region, where both PM_2.5_ and ozone pollution occur simultaneously during the summer season. They found that from 2015 to 2022, the incidence of concurrent PM_2.5_ and ozone pollution during the summer months in the region exceeded fifty percent, with an annual increase observed. Aishan et al. [26] provided statistical insights into the simultaneous occurrence of PM_2.5_ and ozone pollution by analyzing spatial and temporal distribution characteristics in Korla, a city in the northeastern Tarim Basin of China. They showed that the prevailing gusts of wind in spring contributed to the entry of dust particles into the atmosphere, leading to high levels of particulate pollution. In contrast, ozone concentrations reached their peak in summer due to increased temperatures and solar radiation. Wang et al. [10] focused on the Lanzhou area to investigate the co-pollution phenomenon of PM_2.5_ and ozone, especially the effect of enhanced atmospheric oxidizing capacity on these two pollutants. Their research showed that enhanced atmospheric oxidizing power was the primary driver behind increased secondary PM production, aggravating PM_2.5_ and ozone co-polluter concentrations. There is an urgent need to strengthen the synergism in preventing and controlling PM_2.5_ and ozone pollution to reduce the negative influences on the ecological environment and human health that not only threaten public health but pose a crucial obstacle to the sustainable development of society.

## 3. Epidemiological Investigation on Combined Exposure of PM_2.5_ and Ozone

Epidemiology is the scientific discipline that examines the distribution of diseases and health conditions in populations and the factors influencing these patterns. It encompasses the strategies and measures aimed at disease prevention and health promotion. Population-based observational data enable us to systematically analyze the statistical associations between environmental exposures and health outcomes. Epidemiological studies have shown that combined exposure to PM_2.5_ and ozone exacerbates health risks through synergistic effects, and mechanisms of combined pollution are more complex than those of single pollutant and can have wide-ranging and differentiated damaging impacts on multi-age populations and on respiratory, cardiovascular, and other systems.

### 3.1. Mortality

There is a strong association between PM_2.5_ and ozone with mortality risk, indicating a significant synergy between these two pollutants. In a study covering 372 cities, Liu et al. [1] found that when evaluating the combined effects of PM_2.5_ and ozone, their synergistic impact on total mortality exceeded their individual effects. The synergy index was implemented to quantify the synergistic effect of two or more factors acting together [27]. The synergy index was calculated as (RR_11_ − 1)/(RR_01_ − 1 + RR_10_ − 1), where RR was the relative risk of mortality associated with the air pollutants. A synergy index > 1 denoted a synergistic interaction, whereas a synergy index < 1 indicated an antagonistic interaction. They found that PM_2.5_ and ozone had significant synergistic and additive effects on residential mortality, with a synergy index of 1.93. This finding was corroborated by another study, which included data from the cause-of-death surveillance system of Jiangsu Province, China, from 2014 to 2021. The study showed that short-term exposures to particulate matter of different particle sizes (PM_1_, PM_2.5_, and PM_10_) and ozone were associated with mortality with multiplicative and additive interactions. Furthermore, this effect diminished with increasing particle size [28].

Due to the aging population, the effects of combined pollution (especially high-level ozone concentrations) on the health of the elderly are particularly crucial. In severe cases, this can even be fatal. Zhang et al. [29] obtained and analyzed data from 20,352 respondents with an average age of 87.1 years. They found a strong positive correlation between PM_2.5_ and ozone levels and associated mortality in both single- and dual-pollutant models. The mortality rate from PM among individuals aged 85 and older was more than double that of those aged 65 and younger [30]. Furthermore, the death toll among the elderly linked to increasing PM_2.5_ and ozone concentrations was significantly higher [31]. Epidemiological evidence based on cohort studies suggests that exposure to composite pollutants significantly exacerbates health risks through the synergistic effects of multiple pathways. Among them, susceptible groups such as the elderly, children, and patients with underlying diseases show increased respiratory–cardiovascular multi-organ system toxicity and excess mortality, and there is an urgent need to establish targeted environmental health intervention policies.

### 3.2. Respiratory System Diseases

The respiratory tract serves as the main connection between the human body and the external environment. Accumulating evidence has established a strong link between exposure to environmental pollutants, such as PM_2.5_ and ozone, and respiratory diseases, especially in children and the elderly [32,33,34,35]. A study conducted in the United States analyzed respiratory emergency room visits across 869 counties, showing that children had the highest rate of visits (1.94 per 10,000 population). This was followed by older adults at 1.37 and adults at 0.91. These findings suggested that children were more sensitive to environmental pollutants [36]. Another study revealed a pronounced negative impact on lung function among children aged 6–15 years undergoing subchronic exposure to ambient PM_2.5_ (12 µg/m^3^) and ozone (6.7 ppb) [37]. Even living in areas with low pollution—defined as annual average PM_2.5_ concentrations below 12 µg/m^3^ and average yearly ozone concentrations below 45 ppb—still increases the risk of acute respiratory distress syndrome (ARDS) in this population [38]. This further demonstrates the fact that even at lower concentrations, atmospheric pollution can be significantly harmful to specific populations. Xu et al. [39] also demonstrated that an increase in each quartile of daily 8 h maximum ozone concentration and PM_2.5_ was associated with an increased risk of asthma symptoms, indicating PM_2.5_ had a more significant effect in the combined effect, suggesting that it might play a more critical role. The direct impact of ozone on lung cancer mortality is largely unknown. There was a reduction in the risk estimate after adjusting for the effect of ozone compared to the only PM_2.5_ consideration. This implies that ozone might play an intermediary role in lung cancer mortality related to PM_2.5_ [40].

### 3.3. Cardiovascular Diseases

The initiation and progression of cardiovascular disease have a strong correlation with long-term exposure to air pollutants. However, the effects of combined exposure to PM_2.5_ and ozone on cardiovascular health remain unclear. Zhang et al. [41] found that PM_2.5_ and ozone caused cardiac conduction abnormalities. Increased concentrations of PM_2.5_ and ozone were associated with increased electrocardiographic PR Interval, QRS Complex, Corrected QT interval prolongation, and elevated heart rate. In contrast to PM_2.5_, several studies have established that the effects of exposure to ozone on cardiovascular health are uncertain and complicated. For instance, a study covering 400,000 participants found no direct relationship between ozone exposure and cardiovascular mortality when taking into consideration the role of PM_2.5_ [42]. This implied that the effects of ozone on cardiovascular health might be more complicated than PM_2.5_ alone.

### 3.4. Neurological Disorders

PM_2.5_ and ozone are considered to be potential contributors to neurological diseases. Fine particulate matter can penetrate the lungs and traverse the air–blood barrier through respiratory exposure. Once in the blood circulation, it is dispersed to various organs and tissues in the body and even crosses the blood–brain barrier into the brain [43]. Recent studies have found that the transfer of fine particulate matter from the lungs into systemic circulation is achieved by cells acting as carriers [44]. Increased concentrations of particulate matter and ozone have been shown to correlate with stroke mortality, with gaseous pollutants such as ozone serving as significant predictors of acute stroke death when controlling for other confounding factors [45]. A cohort study conducted in Taiwan from 2001 to 2010 involving over 90,000 participants aged 65 and older revealed that the risk of Alzheimer’s disease (AD) significantly increased by 211% (95% CI 2.92–3.33) for every 10.91 ppb increase in O_3_ during the follow-up period, and a 138% (95% CI 2.21–2.56) increase in risk of AD for every 4.34 µg/m^3^ increase in PM_2.5_. These data indicated that pollution levels exceeding EPA standards for both ozone and PM_2.5_ significantly increase the risk of developing AD [46].

### 3.5. Reproductive System Diseases

Much of the previous research on the reproductive effects of air pollutants has focused on the mechanism of individual pollutants. Exposure to PM_2.5_ or ozone increases the risk of preterm labor [47,48,49]. However, there are limited studies specifically addressing the combined effects of combined PM_2.5_ and ozone. Nazeeba et al. [50] found that the combined effects of PM_2.5_ and ozone increased the risk of preterm birth far beyond the impact observed from their individual exposure. Moreover, this combination exposure yielded an adjusted relative risk value of up to 3.63, which was remarkably greater than the relative risks for PM_2.5_ (0.99) or ozone (1.34) alone. A study involving 2212 women in China measured anti-Müllerian hormone (AMH) levels, and discovered a significant correlation between long-term exposures to both PM_2.5_ and ozone and reduced AMH levels in women. The impact of ozone on AMH levels was particularly significant during various stages of follicular development [51]. PM_2.5_ and ozone not only pose potential risks to female fertility and accelerate the aging of the female reproductive system, but they also present a greater risk to pregnancy outcomes, such as preterm labor. These findings not only address the research gap regarding the impact of PM_2.5_ and ozone pollution on the female reproductive system but also provide a more comprehensive and in-depth scientific basis for public health protection and prevention.

## 4. Toxicological Effects of PM_2.5_ and Ozone Combination on Animal Models

This section is based on animal models and systematically evaluates the biological effects of combined exposure to PM_2.5_ and ozone on the respiratory system, cardiovascular system, and cerebrovascular system. This provides a new perspective for developing an early warning biomarker system for climate-sensitive diseases caused by composite pollutants.

### 4.1. Respiratory Toxicity

Many studies have reported respiratory damage from PM_2.5_ or ozone individually, but the toxic effects of combined PM_2.5_ and ozone pollution on the respiratory system under current exposure conditions are of greater concern [52,53,54,55,56,57,58,59,60,61]. Concurrent exposure to PM_2.5_ and ozone exposure induced significant synergistic effects that increased respiratory damage. Compared to ozone exposure, the tracheal drip administration of PM_2.5_ was found to increase total cell counts, inflammatory factors, lactate dehydrogenase (LDH), and total protein in alveolar lavage fluid from rat lung tissues. This finding indicated a worsening of PM_2.5_-induced lung injury, suggesting that the combination of both exposures might have synergistic effects [62]. In another study, combined exposure to ambient particulate matter (CAP) and ozone significantly increased the alveolar lavage fluid levels of N-acetyl-D-aminoglucosidase (NAG), LDH, and lung CuZn-superoxide dismutase (CuZn-SOD) compared to filtered air in winter. No significant changes in NAG and CuZn-SOD levels were observed in the summer exposure groups [63]. This seasonal difference might be attributed to the differences in the composition of particulate matter in different seasons, which in turn affects its toxic effects. These findings provide important clues for understanding the potential harm of compound exposure to air pollution in patients with pre-existing health conditions.

### 4.2. Toxic Effects on the Cardiovascular and Cerebrovascular System

The combined exposure to PM_2.5_ and ozone is one of the triggers for the development of cardiovascular disease. Rats subjected to a high fructose diet and exposed to concentrated ambient fine particulate matter (CAPs at 356 µg/m^3^), ozone (at 0.485 ppm), or a composite of CAPs (at 441 µg/m^3^) and ozone (at 0.497 ppm) showed significant increases in epicardial adipose (EAT) and perirenal adipose tissue (PAT). In comparison to exposure to a single pollutant, co-exposure resulted in a significantly greater accumulation of these adipose tissues. There was a significant increase in the expression of inducible nitric oxide synthase (iNOS) protein, along with a decrease in mitochondrial area in the EAT and PAT. These alterations collectively suggested that composite exposure might adversely affect cardiovascular health [64]. Wang et al. further found that Wistar rats exposed to ozone (at a concentration of 0.81 ppm) and different doses of PM_2.5_ (0.2, 0.8, or 3.2 mg/rat, respectively) showed that exposure to PM_2.5_ alone significantly increased C-reactive protein (CRP), malondialdehyde (MDA), and creatine kinase (CK) in mice, while heart rate variability (HRV) decreased. Ozone exposure alone did not cause any adverse effects. However, combined exposure to PM_2.5_ and ozone significantly exacerbated these adverse effects, indicating that ozone enhanced the damage to the heart and overall function of mice caused by PM_2.5_ [65].

## 5. Mechanism of PM_2.5_ and Ozone-Induced Damage

This section systematically elucidates the toxicity effect network of PM_2.5_ and ozone co-exposure utilizing animal models and multi-scale in vitro systems. These findings provide critical theoretical support for the establishment of a precise exposure warning system based on compound pollutant metabolic fingerprints, and the development of novel detoxifiers for targeted therapy and prevention.

### 5.1. Impact of Ozone on the Physicochemical Properties of PM_2.5_

PM_2.5_ and ozone are co-existent pollutants in the natural environment. Both PM_2.5_ and ozone share common precursors, such as NO_x_ and VOCs, and they interact with each other in the atmosphere through multiple pathways [66,67]. As a strong oxidant, ozone causes the formation or degradation of both organic and inorganic secondary aerosols through non-homogeneous reactions occurring on the surface of particulate matter, free-radical chain reactions, and other reactions with reactive components and precursors associated with PM_2.5_ [68,69,70]. Different sources of PM_2.5_, as listed in Table 1, have different mechanisms of interaction with ozone. Ma et al. [71] analyzed fresh PM_2.5_ from different sources, including suburban areas, cooking emissions, motor vehicle emissions, industrial areas, and biomass combustion sources, and examined the characteristics of PM_2.5_ following ozone aging. They found that the aging process reduced the oxidation potential (OP) of PM_2.5_ from all sources and that ozone-induced alterations in the hygroscopicity and phase state of PM_2.5_ by modifying its chemical composition. One of the main components of PM_2.5_, black carbon (BC), underwent a series of changes on its surface after exposure to ozone. This process led to an increase in organic carbon components, such as ketones and lactones on the surface of BC, as well as the formation of oxygen-containing functional groups including carbonyls and hydroxyls. These changes increase the oxidative properties, water absorption, and polarity of particulate matter surfaces. The electron paramagnetic resonance (EPR) signals of ozone-oxidized BC were significantly enhanced, accompanied by the generation of free radicals [72]. This observation revealed the significant effect of ozone on the physicochemical properties of BC. Another study found that the levels of polycyclic aromatic hydrocarbons (PAHs) in biodiesel exhaust decreased by 3–4 fold following exposure to ozone [73]. Due to the variety of components and the complex secondary substances generation and transformation between PM_2.5_ and ozone, understanding the mechanisms of compound toxicity has become an important issue in the field of environmental pollution. Further studies need to pay more attention to the inter-transformation mechanism between PM_2.5_ and ozone and their toxicological effects on living organisms.

### 5.2. Damage to Biological Barrier Induced by the Interaction Between PM_2.5_ and Ozone

Ozone is an essential oxidant in the atmosphere and interacts with PM_2.5_ complexly during atmospheric aging [74]. Both pollutants pose significant threats to the biological barrier, each inducing damage through different mechanisms. However, under certain environmental conditions, they may synergistically interact and amplify their detrimental effects on membrane integrity. BC undergoes a photochemical oxidation process to form aged BC particles, known as oxidized BC (OBC). After treatment with A549 cells with 5, 10, 20, and 40 µg/mL of OBC, respectively, the mitochondrial membrane potential was significantly increased in a dose-dependent way. This alteration led to the hyperpolarization of the mitochondrial membrane potential, which compromised mitochondria integrity and initiated the mitochondria-mediated apoptosis pathway [75]. Cui et al. [76] found that macrophages underwent necrotic apoptosis and cell membrane rupture following exposure to OBCs. Furthermore, the damage to the biological barrier was exacerbated by significant increases in reactive oxygen species (ROS) and cytoplasmic calcium ion levels. Regarding animal experiments, the study conducted by Nairrita et al. [77] showed that combined exposure to PM_2.5_ and ozone had destructive effects on the biological barrier. The protein content and LDH activity in alveolar lavage fluid (BALF) were significantly increased following composite exposure to CB and ozone compared to control groups exposed to CB alone and ozone alone. These findings suggested that the lung–blood barrier was damaged, ultimately leading to lung cell death.

### 5.3. Inflammatory Response Induced by the Combination of PM_2.5_ and Ozone

The inflammatory response is an important defense mechanism for the body in the face of injury, infection, or noxious stimuli. Although numerous studies have explored the mechanisms of inflammatory damage when PM_2.5_ and ozone act alone [78,79,80,81,82,83,84,85], their effects become more complicated when PM_2.5_ and ozone co-exist. In such cases, their interaction is not merely additive; rather, they may exhibit intricate synergistic or potentiating effects.

The study by Wang et al. [86] found that ozone augments PM_2.5_-induced inflammatory responses. Compared to controls and rats solely exposed to PM_2.5_, those subjected to both PM_2.5_ (3.2 mg/rat) and ozone exhibited more severe lung inflammation in rats, including significantly elevated neutrophil counts in alveolar lavage fluid and tumor necrosis factor-α (TNF-α) levels. Another study performed a co-exposure experiment with ovalbumin (OVA)-allergic mice. The combined exposure to both pollutants significantly worsened allergic asthma in a dose-dependent manner, leading to increased airway hyperresponsiveness, the presence of astrocytic chemotaxis, more severe airway inflammation, and higher levels of oxidative stress as compared to exposure to PM_2.5_ or ozone alone. Moreover, the combined exposure caused transient receptor potential vanilloid 1 (TRPV1) expression and substance P (SP) production and elevation, further exacerbating lung inflammation caused by neurogenic inflammation [87]. This finding revealed that the combined effects of ozone and PM_2.5_ were more harmful to lung health than the effects of exposure alone.

These findings were further corroborated by clinical studies. In a study performed in China, exhaled breath condensate (EBC), peripheral blood, and urine were collected from 108 non-smoking 50–65-year-olds and analyzed for 1327 compounds in an individual PM_2.5_ exposure profile. The results showed that ozone increased the expression of PM_2.5_-induced pro-inflammatory markers, such as exhaled nitric oxide, serum IL-6, IL-1β, TNF-α, and malondialdehyde. It was also found that exposure to ozone enhanced respiratory and systemic inflammatory responses to organic compounds in PM_2.5_ through the physiological pathways of cytochrome P450 (CYP450) and NRER. Aromatic compounds and terpenoids were identified as the main constituents of the ozone-enhanced inflammatory effects associated with PM_2.5_ [88]. A study by Kurai et al. [89] further confirmed the synergistic effect between ozone and particulate matter. They found that when THP1 cells were exposed to both particulate matter (particle size less than 1 µm) and ozone, ozone was able to significantly increase particulate matter-induced interleukin-8 production compared to exposure to particulate matter alone or solvent only. These findings suggested that ozone might enhance the ability of particulate matter to induce inflammatory responses by altering its chemical composition. In contrast, the antioxidant acetylcysteine (NAC) significantly inhibited the particulate matter-induced increase in IL-8, suggesting that ozone enhanced the ROS-generating pathway by altering the particulate matter chemistry.

### 5.4. Oxidative Stress Induced by the Combination of PM_2.5_ and Ozone

Oxidative stress refers to an imbalance between oxidative and antioxidant processes in cells and tissues, resulting in the generation and accumulation of oxygen-active substances, such as ROS, causing damage to lipids, proteins, and DNA [90,91,92]. The lung is one of the major target organs of PM_2.5_ and ozone, and oxidative stress is one of the main mechanisms of lung damage caused by PM_2.5_ and ozone.

The researchers found that exposure of A549 cells to both PM and ozone resulted in a significant increase in intracellular PM deposition, a significant increase in 4-NHE levels, and an increase in nuclear Nrf2 levels compared to exposure to PM alone [93]. This combination of factors subsequently contributed to the promotion of oxidative stress. Malondialdehyde (MDA) is a typical oxidative stress biomarker. The researchers collected nasal fluid from 43 asthmatic children aged 5 to 13 years and assessed asthma symptoms using the Children’s Asthma Control Test (C-ACT). They measured PM_2.5_ and ozone concentrations in the children’s bedrooms and outside their windows. The findings showed that the nasal MDA concentrations increased, and the C-ACT scores decreased with prolonged exposure, suggesting that oxidative stress played an important role in the association between air pollutants and respiratory disease [94].

As shown in Table 1 and Table 2, the physicochemical properties of PM_2.5_ are influenced by the intricate processes involved in ozone generation and transformation. Most studies focused on how variations in ozone levels affect the physicochemical properties of PM_2.5_, including PH value, hygroscopicity, and particle size. In terms of compounded toxicity effects, a significant number of studies investigated the synergistic interactions between PM_2.5_ and ozone on the mechanisms of inflammatory response and oxidative stress. Using different model organisms (e.g., A549 cell, RAW264.7 cell, and Wistar rats), different inflammatory factors and oxidative stress markers were investigated. The findings indicated that PM_2.5_ and ozone exhibit synergistic effects; however, these interactions are affected by a variety of factors, such as individual differences, exposure concentration, and exposure time. Collectively, these studies not only enriched our understanding of the impact of ozone on inflammation, but also contributed to a more comprehensive body of research regarding its synergistic effects with PM_2.5_ at a mechanistic level.

## 6. Conclusions

Based on multicenter cohort analysis and toxicity mechanism experiments, the combined exposure of PM_2.5_ and ozone can produce significant synergistic health effects. In vivo and in vitro studies have demonstrated that ozone co-exposure amplifies PM_2.5_-induced oxidative stress, inflammatory processes, loss of membrane integrity, and reproductive dysfunction. These findings provide key dose–response evidence for revising the framework of health risk assessment related to air pollution and contribute to bridging the theoretical gap in understanding multi-pollutant interaction mechanisms.

## 7. Implications and Prospect

The synergistic damage effects of combined exposure to PM_2.5_ and ozone, as revealed in this study, provide critical scientific evidence for the need for integrated treatment approaches addressing both pollutants. While the existing research has drawn macroscopic toxicological results for the co-exposure of PM_2.5_ and ozone, there are still critical knowledge gaps in elucidating the molecular toxicological mechanisms of composite exposure. The introduction of new technologies and detection methods is urgently required. For example, using real-time dynamic monitoring techniques can facilitate tracking PM_2.5_ and ozone co-exposure in living biological models, allowing us to observe the accumulation of PM_2.5_ in organs and tissues over time. We can more accurately assess the relationship between combined exposure to PM_2.5_ and ozone and disease risk by analyzing metabolites in exhaled breath through respiratory genomics. Using integrated genomics, proteomics, metabolomics, and other bioinformatics tools will help to identify specific targets of PM_2.5_ and ozone and ultimately elucidate the molecular mechanisms of the combined pollutants. These studies will not only contribute to a more comprehensive understanding of the toxic effects of PM_2.5_ and ozone, but they will also provide a scientific basis for formulating more effective environmental protection policies and health protection measures. These findings emphasize the urgent need to revise the current risk assessment framework and consider the synergistic effects of multiple pollutants in environmental health policies. Furthermore, based on the synergistic health effects of PM_2.5_ and ozone-sensitive populations such as the elderly, children, and pregnant women, a multidimensional protection system will be constructed to achieve effective prevention and treatment.

## Figures and Tables

**Table 1:** The influence of ozone on the chemical composition of PM_2.5_.

Source of Particulate Matter	Changes in Physical and Chemical Properties	Mechanism	References
PM_2.5_	OPs; hygroscopicity	Ozone affects the properties and toxicity of PM_2.5_ by modifying the reactive organic compounds in it.	[71]
BC	pH values; hygroscopicity, oxygen-containing functional groups; water-soluble organic matter; free radicals.	Ozone changes the physicochemical properties of BC through oxidation, thereby increasing its toxicity.	[72]
Biodiesel particulate matter	All PAHs	Ozone reacts with PAH in Biodiesel particulate matter, where the C=C double bond may provide a reactive site for ozone attack.	[73]

**Table 2:** Toxicological mechanisms of combined PM_2.5_ and ozone exposure.

Research Object	Exposure Mode	Toxic Effect	References
A549 cell	OBC: Co-incubation (5, 10, 20, and 40 µg/mL)	OBC exposure significantly increased ROS levels, altered mitochondrial permeability, and disrupted mitochondrial membrane integrity in A549 cells.	[75]
RAW264.7 cell	OBC: Co-incubation (50 and 100 mg/L)	Exposure to OBC exacerbates cell membrane rupture by significantly upregulating reactive oxygen species (ROS) and cytoplasmic calcium ion levels.	[76]
C57BL/6J male mice	CB: Inhalation (10 mg/m^3^) Ozone: Inhalation (2 ppm)	Combined CB and ozone exposure mediates disruption of the lung–blood barrier via epithelial alertin.	[77]
Wistar rats	PM_2.5_: tracheal drip (0, 0.2, 0.8, and 3.2 mg/Rat) Ozone: inhalation (0.8 ppm)	Ozone enhances PM_2.5_-induced inflammatory responses.	[86]
Balb/c mice	PM_2.5_: tracheal drip (0.5 mg/mL) Ozone: inhalation (0.5 ppm)	Combined exposure to PM_2.5_ and ozone has a synergistic effect on allergic inflammation.	[87]
Human	108 non-smokers aged 50–65 years old	Synergistic enhancement of PM_2.5_-induced inflammation by ozone	[88]
THP1 cell	PM_2.5_: co-incubation (10 µg/mL) Ozone: co-incubation (0.12 and 0.24 ppm)	Significant synergistic effects of ozone and PM_2.5_ on IL-8 production	[89]
A549 cell	PM_2.5_: co-incubation (1 µg/mL) Ozone: co-incubation (0.1 ppm)	Combined ozone and PM exposures act synergistically in enhancing oxidative stress-induced cellular damage.	[93]
Human	inhalation through the respiratory tract	PM_2.5_ and ozone exposure increase nasal oxidative stress marker MDA concentrations	[94]

## Data Availability

All the datasets generated for this study are included in the article. In this study, the design of the topic, the reference research, and the analysis of the results were all performed independently by the authors, and AI was only used for language touch-ups (grammar proofreading and sentence adjustment). No opinion generation or data interpretation was involved. The final draft of the article was screened by Turnitin AI detection system. The detection report meets the journal’s requirements for original work.
